# Antibodies against *Borrelia burgdorferi* Sensu Lato in Clinically Healthy and Sick Horses: First Report from the Czech Republic

**DOI:** 10.3390/microorganisms11071706

**Published:** 2023-06-29

**Authors:** Nikola Kašpárková, Eva Bártová, Alena Žákovská, Marie Budíková, Kamil Sedlák

**Affiliations:** 1 Department of Biology and Wildlife Diseases, Faculty of Veterinary Hygiene and Ecology, University of Veterinary Sciences Brno, Palackého tř. 1946/1, 612 42 Brno, Czech Republic; kasparkova.nicola@seznam.cz; 2 Department of Experimental Biology, Faculty of Science, Masaryk University, Kamenice 5, 625 00 Brno, Czech Republic; alenazak@sci.muni.cz; 3 Department of Biology, Faculty of Education, Masaryk University, Poříčí 7/9, 625 00 Brno, Czech Republic; 4 Department of Mathematics and Statistics, Faculty of Science, Masaryk University, Kotlářská 267/2, 611 37 Brno, Czech Republic; budikova@math.muni.cz; 5 State Veterinary Institute Prague, Sídlištní 136/24, 165 03 Prague, Czech Republic; kamil.sedlak@svupraha.cz

**Keywords:** borreliosis, equine, ELISA, Lyme diseases

## Abstract

Lyme disease, caused by some strains of bacterial spirochetes *Borrelia burgdorferi* sensu lato (*Bb*sl), affects humans but also domestic animals including horses. The primary pathogens in horses in Europe are *B. afzelii*, *B. garinii* and *B. burgdorferi* sensu stricto. To our knowledge, there are no data available on the seropositivity of *B. burgdorferi* s.l. in horses from the Czech Republic. In this country, horses are mainly used for sport, breeding, and recreational riding in areas where vectors of *B. burgdorferi* s.l. are present, which is why they are frequently at risk of infection. The aim of the study was to detect anti-borrelia IgM and IgG antibodies in clinically healthy and sick horses from the Czech Republic and to evaluate the risk factors of infection. In total, sera of 262 horses (247 clinically healthy horses and 15 horses hospitalized due to symptoms of encephalitis/meningoencephalitis) were examined by an indirect sandwich enzyme-linked immunosorbent assay. Positivity of *B. burgdorferi* was 27% (66/247) in clinically healthy horses (21% IgM, 7% IgG and 3% IgM + IgG antibodies) and 20% (3/15) in horses with clinical signs (20% IgM, 7% IgG and 7% IgM + IgG). In the clinically healthy horses, positivity statistically differed (*p* ≤ 0.05) only in Pony and Warmblood breeds, being the most affected at 32% and 30%, respectively, while other characteristics (sex, age, usage and localities) had no effect on positivity. This is the first survey of antibodies to *B. burgdorferi* s.l. in Czech horses showing that horses are exposed to ticks infected with *B. burgdorferi* s.l. This should be taken into account when making differential diagnoses in patients with non-specific symptoms to start with adequate therapy.

## 1. Introduction

Lyme disease is serious infection affecting the skeletal, cardiovascular and nervous system that can lead to erosive arthritis of larger joints, cardiomyopathy, atrioventricular blocade, meningitis or cranial neuritis [1,2]. This disease is caused with the Gram-negative bacteria belonging to the spirochetes and *Borrelia burgdorferi* sensu lato (*Bb*sl) families. Transmission of infection occurs through ticks and varies depending on the species of ticks and several other factors such as the vector’s density and activity, abundance of reservoir animals and other blood hosts, land cover and human behavior and is also influenced by the weather and climate [3]. In Europe, *Ixodes ricinus* is a well-known blood feeding vector of *B. burgdorferi* s.l. with a high incidence and three host life cycles (lasting 2–6 years) that favor the potential spread of many pathogens, including *Bb*sl. When the larval stage hatches from an egg, it first starts feeding typically on a small rodent or bird and then transforms into the nymphal stage. After the second feeding, usually on small or bigger mammals, it performs its final transformation into adulthood and is capable of mating. Only females feed on blood and can lay 500–2000 eggs on the vegetation [4,5,6]. Since the 1980s, the tick vectors of this infection have increased in density and spread in Europe into areas of higher latitudes and altitudes. It can be assumed that climate change in Europe will facilitate the spread of infection and the increased occurrence of disease in new areas [7]. In contrast, in some areas where climatic conditions become too hot and dry for tick survival, Lyme disease may disappear [8].

In horses, this disease is usually asymptomatic, but there were also cases with clinical signs including meningitis, cranial neuritis, radiculoneuritis, sporadic lameness, laminitis, swollen joints, muscle tenderness and weight loss [9,10]. In Europe, antibodies to *B. burgdorferi* s.l. were detected, e.g., in 48% of horses from Slovakia [11] and in 26% of horses from Poland [12]. To our knowledge, there are no data available on the seropositivity of *B. burgdorferi* s.l. in horses from the Czech Republic. In this country, horses are mainly used for sport, breeding, and recreational riding in areas where vectors of *B. burgdorferi* s.l. are present, which is why they are frequently at risk of infection. For example, there was a three-year research study (2011–2013) on the population density of *I. ricinus* conducted in four topographically distant areas (the areas of the Ústí nad Labem Region, Olomouc Region, South Bohemian Region, and Highlands Region) in the Czech Republic [13]. Spirochetes from *B. burgdorferi* s.l. complex were detected in all studied sites in all altitudes from 280 to 1030 m a. s. l., with a total rate of infection 11.4%. Three of the medically most important *Borrelia* species (*B. afzelii*, *B. garinii* and *B. burgdorferi* s.s.) formed a core group among all detected genospecies. In the Moravian regions of the Czech Republic, *B. burgdorferi* s.l. was detected in 12% [14]—26% [15] of collected ticks.

The aim of this study was to estimate the seropositivity of *B. burgdorferi* s.l. in clinically healthy and sick horses from the Czech Republic and to evaluate the risk factors of infection.

## 2. Materials and Methods

### 2.1. Samples

In total, 262 blood samples were collected from the *vena jugularis* of horses between 2008 and 2014 and were stored at −20 °C before being used for the serological detection of antibodies to *B. burgdorferi* s.l. The samples were divided into two groups. The first group included sera from 247 clinically healthy horses, older than 1 year, that spent a lot of time in the pastures and were used for breeding, recreational riding, or sport. The second group included sera from 15 horses with clinical symptoms of polysynovitis (*n* = 1), tetraparesis (*n* = 1), myeloencephalitis (*n* = 1), arhtropathies (*n* = 1) and other non-specific symptoms of tick-borne Lyme diseases (*n* = 11). The sick horses were hospitalized for clinical symptoms and treatment at the Equine Clinics. The characteristics of the horses, including sex, age, breed, usage, and localities are summarized in Table 1 for healthy horses and in Table 2 for sick horses. Owners of serologically positive horses were interviewed to obtain the necessary information about the horses.

### 2.2. Detection of Antibodies to Borrelia burgdorferi s.l.

The samples were examined for *B. burgdorferi* s.l. antibodies by the modified enzyme-linked immunosorbent assay (ELISA, TestLine, Brno, Czech Republic). A mixture of ultrasonically disrupted whole cell antigens of *Borrelia afzelii* isolated from *Ixodes ricinus* (Switzerland VP 13/12, Bioveta a.s. Ivanovice na Hané, Czech Republic), *Borrelia garinii* isolated from *I. ricinus* (France VP 14/12, Bioveta a.s.) and *Borrelia burgdorferi* s.s. isolated from human (VP n. 19/12, Bioveta a.s.) was diluted with carbonate buffer at pH 9.6 (2 µg/mL) and incubated on microplates overnight at 4 °C. It was then washed three times with phosphate buffer (pH 7.4) containing 0.05% of Tween 20 (washing solution). After that, 100 µL of sera (diluted at 1:100 in phosphate buffer with washing solution and 0.3% casein as binding solution) was inoculated into microplates and incubated at 37 °C for 1 h. After the triple washing of the microplates, 100 µL of anti-horse IgM or IgG peroxidase conjugates (Sigma-Aldrich spol. s.r.o., Prague, Czech Republic), diluted with a binding solution at 1:4000 and 1:3000, respectively, were added per well and incubated at 37 °C for 1 h. After subsequent washing with the washing solution, 100 µL of substrate solution (0.1 M citrate buffer pH 4.7–5.0 with 0.05% H_2_O_2_) with orthophenylene diamine was added per well. The reaction was stopped after 20 min of incubation by adding one 1M H_2_SO_4_. The sera of horses positive and negative for IgM and IgG antibodies to *B. burgdorferi* s.l. (Bioveta a.s.), were used as positive and negative controls, respectively. The absorbance was measured at 492 nm on a spectrophotometer (SLT RainBow, Schoeller instruments, s.r.o., Prague, Czech Republic). Cluster analysis with the K-diameter method [16] was used for the evaluation of IgM and IgG positive, dubious, and negative results. Cluster analysis was applied to the data plotted in sub-graphs. Samples were marked as positive, when IgM antibodies (samples with absorbance ≥ 0.54), IgG antibodies (samples with absorbance ≥ 0.79) or both IgM and IgG antibodies were detected.

**Table 1:** Characteristics of clinically healthy horses tested for antibodies (IgG and IgM) to *Borrelia burgdorferi* sensu lato by the modified enzyme-linked immunosorbent assay.

Charactistics	Total Number	IgG	IgM	IgG + IgM	Total	*p*-Value
		Positive (%)	Positive (%)	Positive (%)	Positive (%)	
Sex						0.0700
Male (stallions and geldings)	82	6 (7%)	16 (20%)	3 (4%)	19 (23%)	
Female	161	12 (7%)	37 (23%)	5 (3%)	44 (27%)	
Not known	4	1 (25%)	2 (50%)	-	3 (75%)	
Age						0.1576
≥3 years	41	-	6 (15%)	-	6 (15%)	
<3 years	203	18 (9%)	49 (24%)	8 (4%)	59 (29%)	
Not known	3	1 (33%)	-	-	1 (33%)	
Breed **						0.0373 *
Arabian horse	9	-	-	-	-	
Coldblood	13	-	1 (8%)	-	1 (8%)	
Pony	25	-	8 (32%)	-	8 (32%)	
Thoroughbred	23	-	3 (13%)	-	3 (13%)	
Warmblood	172	18 (10%)	41 (24%)	8 (5%)	51 (30%)	
Not known	5	1 (20%)	2 (40%)	-	3 (60%)	
Use						0.7012
Pasture	83	7 (9%)	18 (22%)	2 (2%)	23 (28%)	
Recreational riding	142	12 (8%)	33 (23%)	6 (4%)	39 (27%)	
Sports	21	-	4 (19%)	-	4 (19%)	
Working	1	-	-	-	-	
Locality (region)						0.3171
Central Bohemia	28	1 (4%)	7 (25%)	-	8 (29%)	
Hradec Králové	11	-	2 (18%)	-	2 (18%)	
Karlovy Vary	12	-	5 (42%)	-	5 (42%)	
Liberec	14	-	4 (29%)	-	4 (29%)	
Moravian-Silesian	33	-	5 (15%)	-	5 (15%)	
Olomouc	22	2 (9%)	5 (23%)	-	7 (32%)	
Pardubice	30	9 (30%)	7 (23%)	4 (13%)	12 (40%)	
Southern Moravia	57	7 (12%)	9 (16%)	4 (7%)	12 (21%)	
Ústí nad Orlicí	6	-	1 (17%)	-	1 (17%)	
Vysočina	24	-	5 (21%)	-	5 (21%)	
Zlín	10	-	5 (50%)	-	5 (50%)	
Total	247	19 (8%)	55 (22%)	8 (3%)	66 (27%)	

* *p* value ≤ 0.05, which represents a statistically significant difference. ** Arabian Horses included Anglo-Arabian Horse (2), Arabian Horse (9), Shagya Arab (1); Coldblood Horses included Brabant (2), Noriker Horse (11); Pony Horses included Danish Sport Pony (1), Hucul Pony (5), Pony of the Americas (11), Shetland Pony (1), Welsh Pony (7); Thoroughbred Horses included Thoroughbred (23); Warmblood Horses included American Paint horse (1), American trotter (3), Andalusian Horse (1), Belgian Warmblood Horse (18), Budyonny Horse (1), Czech Warmblood (109), Fjord Horse (1), Friesian Horse (2), Haflinger (3), Hannoverian Horse (1), Karachay horse (3), Kladruber (16), Polish Warmblood Horse (1), Silesian Horse (6), Slovakian Warmblood Horse (5), Wielkopolski (1).

**Table 2:** Detailed characteristics of horses with symptoms of borreliosis tested for antibodies to *Borrelia burgdorferi* sensu lato.

Characteristics	Total	IgG	IgM	IgG + IgM	Total	*p*-Value
		Positive (%)	Positive (%)	Positive (%)	Positive (%)	
Sex						0.4843
Male (stallions and geldings)	7	1 (14%)	2 (29%)	1 (14%)	2 (29%)	
Female	7	-	1 (14%)	-	1 (14%)	
Not known	1	-	-	-	-	
Age						0.4843
≥3 years	7	1 (14%)	2 (29%)	1 (14%)	2 (29%)	
<3 years	7	-	1 (14%)	-	1 (14%)	
Not known	1	-	-	-	-	
Breed *						
Arabian horse	3	-	-	-	-	
Coldblood	2	-	1 (50%)	-	1 (50%)	
Thoroughbred	4	1 (25%)	2 (50%)	1 (25%)	2 (50%)	
Warmblood	6	-	-	-	-	
Use						
Pasture	3	-	2 (67%)	-	2 (67%)	
Recreational riding	4	-	-	-	-	
Sports **	3	1 (33%)	1 (33%)	1 (33%)	1 (33%)	
Young horse ***	3	-	-	-	-	
Not known	2	-	-	-	-	
Locality (region)						
Central Bohemia	3	1 (33%)	1 (33%)	1 (33%)	1 (33%)	
Olomouc	2	-	1 (50%)	-	1 (50%)	
Pardubice	1	-	-	-	-	
Praha	1	-	-	-	-	
Southern Moravia	5	-	1 (20%)	-	1 (20%)	
Western Slovakia	1	-	-	-	-	
Not known	2	-	-	-	-	
Total	15	1 (7%)	3 (20%)	1 (7%)	3 (20%)	

* Arabian Horses included Arabian horse (3); Coldblood Horses included Irish Cob (2); Thoroughbred Horses included Thoroughbred (4); Warmblood Horses included Czech Warmblood (3), Friesian horse (1), Russian Trakehner (1); ** Sports included racing (1), endurance riding (1) and others (1); *** Young Horses included sucking foal (1), foal (1) and horses aged up to 3 years (1).

Moreover, twenty-two randomly selected sera were used to confirm the results of ELISA (positive, borderline, and negative) in individual *B. burgdorferi* strains (*B. afzelii*, *B. garinii*, and *B. burgdorferi* s.s.).

### 2.3. Statistical Analysis

The results were statistically analyzed with Pearson’s chi-square test for independence using STATISTICA Cz 12 [17]. We tested the null hypothesis that the positivity of *B. burgdorferi* s.l. was independent of other categorial variables such as sex, age, breed, usage of horses, and localities. This null hypothesis was rejected when the *p*-value was <0.05. In the case of a statistically significant difference in some of the variables, the Scheffé multiple comparison method [17] was subsequently applied in order to compare estimates among all levels of the categorical variables.

## 3. Results

Antibodies to *B. burgdorferi* s.l. were detected in 66 (27%) of 247 clinically healthy horses (Table 1). Antibodies to IgM were detected in 55 (22%) horses and IgG in 19 (8%) horses; antibodies to both IgM and IgG were detected in 8 (3%) horses. Positivity statistically differed among breeds (*p* = 0.0373) of horses, but Scheffé’s method of multiple comparison did not find differences between pairs of breeds. Other characteristics such as sex, age, usage, and locality had no effect on seropositivity (Table 1).

Antibodies to *B. burgdorferi* s.l. were detected in 3 (20%) out of 15 horses with clinical symptoms (Table 2), without a statistical difference (*p* ≥ 0.05) in sex and age. The breed, usage and locality factors were not statistically evaluated, due to too low frequencies in some categories. In Table 3, the details of four seropositive horse patients (three horses positive in ELISA and also one horse negative in ELISA, but positive in Immunoblot) are presented to show their history, diagnosis, and treatment.

Moreover, the results of the ELISA (positive, borderline, and negative) in individual *B. burgdorferi* strains (*B. afzelii*, *B. garinii*, and *B. burgdorferi* sensu stricto) were confirmed by testing 22 randomly selected blood sera. Antibodies against at least two of the above-mentioned antigens were proved in all 22 samples, with *B. burgdorferi* ss being the most common, followed by *B. afzelii* and *B. garinii.*

## 4. Discussion

Results from our study showed that horses in the Czech Republic are exposed to ticks infected with *B. burgdorferi* sensu lato with 27% positivity in clinically healthy horses and 20% in horses with clinical symptoms. The positivity obtained in healthy horses from our study was similar, with 29% in horses from Denmark [18], 26% in horses from Poland [12] and 24% in horses from Italy [19]. In contrast, a higher positivity was detected in horses from Brazil [20], with a prevalence of 58.3%, and in horses from the USA [21], with a prevalence of 75%. On the other hand, lower positivity rates were observed in horses from Romania [22], with a prevalence of 12%, and in horses from Korea [23], with a prevalence of 5.5%. IgM antibodies are detectable during the acute form of infection for a short time (for weeks and months), compared to IgG antibodies detectable for a long time during chronic infection. Ponies, experimentally infected with *B. burgdorferi* and untreated, remained infected with live culturable organisms 9 months later, confirming that chronic infection is possible in horses [24]. Many horses, including those that received antimicrobial treatments for *B. burgdorferi*, had positive serological tests for several months or even years. Our results may indicate that horses with IgM antibodies were exposed to *B. burgdorferi* s.l. in spring or in early summer, while those with IgG antibodies were infected a longer time before. The higher positivity for IgM antibodies compared to IgG antibodies could also be explained by the higher sensitivity of ELISA to IgM antibodies.

Females had a higher positivity (27%), while males (stallions and geldings) had a slightly lower positivity (23%) and, additionally, there was a higher positivity in older horses (29%) compared to younger horses (15%). The higher positivity in females in our study can be attributed to their increased exposure to ticks in pastures, primarily due to their use in breeding. In contrast, males, commonly used for sports, have less frequent exposure to tick-infested areas. Our findings align with previous studies [22,23,25] that also found no correlation between positivity and age or sex, supporting our hypothesis. Positivity statistically differed between breeds of horses but Scheffé’s method of multiple comparison did not find differences between pairs of breeds. This paradox can be explained by the fact that Pearson’s chi-square test can detect an overall statistically significant difference between categories, but Scheffé’s method is not sensitive enough to detect more minor differences between specific pairs of categories. The highest positivity was in the Pony (32%) and Warmblood (30%) breeds. Similar results were proved by Lee et al. [23], who described a statistically significant difference between breed and region in their studied horses and also Laamari et al. [25], who described crossbred animals to be more infected with *B. burgdorferi*. In contrast, Štefančíková et al. [12] did not prove any difference in positivity between breeds of horses. The positivity in horses grazing in pastures or used for recreational riding had a higher level of antibodies compared to horses used for sport, but without a statistical difference. Similarly, Egenvall et al. [26] and Kiss et al. [22] did not find an effect of the usage of horses on seropositivity. However, there may be an association between the breed and usage of horses because racehorses are probably less exposed to ticks compared to working horses [26]. We can assume that there could be different seropositivity in the same horse breeds kept in different environments. This can be attributed to the ecology of vector ticks and the susceptibility of the hosts [23]. However, in our study, the positivity of *B. burgdorferi* did not differ in horses from different localities (regions). Similar results were also obtained in horses from Slovakia [11], Sweden [26], and Italy [19].

Seropositivity does not necessarily mean that the disease is going to develop with clinical signs, because only 5–10% of seropositive horse individuals develop clinical symptoms [18,27]. Horses, as long-living animals, are more commonly exposed to the infection without clinical signs, but they may still be carriers of infection [28]. In our study, antibodies to *B. burgdorferi* s.l. were detected in only 20% (3/15) of horses with clinical symptoms. This is in accordance with other studies, where animals with clinical symptoms of Lyme disease were often seronegative [29,30]. It is important to consider that clinical symptoms observed in Lyme disease can be due to other reasons such as viral or bacterial infections, toxic diseases, or can be the result of other inflammatory processes. This fact can explain why animals with clinical symptoms of Lyme disease can often be seronegative. Horses in our study were hospitalized for symptoms of encephalitis, meningoencephalitis and arthropathies associated with movement disorders. These symptoms corresponded to the clinical signs of the nervous form of Lyme disease, as was described in horses from the USA [29,31]. For example, horse patient no. 1 had a history of painful joint swelling of all the limbs, lasting for 14 days without reaction to the therapy, limping and fever (38.8–39.4 °C). All these clinical symptoms could correspond to Lyme disease, as described in many studies e.g., [32]. The first serological examination of horse performed by ELISA in 2009 was negative for *B. burgdorferi* s. l. antibodies, but the second examination in 2013 by immunoblot (on three different microtiter plates) was positive, confirming Lyme disease. This case confirmed the need for a comprehensive diagnosis based on more diagnostic methods in the case of suspected Lyme disease, as suggested by Shapiro [33].

In our study, 22 selected blood sera were tested to confirm the results of ELISA (positive, borderline, and negative) in individual *B. burgdorferi* strains (*B. afzelii*, *B. garinii*, and *B. burgdorferi* sensu stricto). Antibodies against at last two of the above-mentioned antigens were proved in all 22 samples, with *B. burgdorferi* ss being the most common, followed by *B. afzelii* and *B. garinii.* Štefančíková et al. [12] found the most common antibodies in horses against *B. afzelii*, then *B. burgdorferi* ss and, lastly, *B. garinii*. Similar results, with the most frequent being *B. afzelii*, followed by *B. garinii* and *Bb*ss, were also reported in ticks from the Czech Republic [34]. To obtain more accurate information on the positivity of antibodies against these antigens, a much larger number of samples would need to be investigated. Many studies have also reported other *B. burgdorferi* s.l. strains to be pathogenic [35,36]. Nevertheless, strains such as *B. bavariensis*, *B. bissettii* and *B. spielmanii* are not routinely tested in laboratories yet. This fact may partly justify the absence of antibodies in horse patients with clinical signs of borreliosis.

## 5. Conclusions

Lyme disease is the most common vector-borne disease in Europe, with the highest incidence reported in Austria, the Czech Republic, Germany, and Slovenia [7]. Results from this study showed that horses in the Czech Republic are exposed to ticks infected with *B. burgdorferi* sensu lato. It is therefore advisable to take this into account when making differential diagnoses in patients with non-specific symptoms, so that adequate therapy will be started in time. Lyme disease is a disease to which horse owners and veterinarians in the Czech Republic should pay attention. As a preventive measure, it is advisable to carry out regular inspections of horses with the timely removal of any ticks and to use acaricidal products or repellents.

## Figures and Tables

**Table 3:** History, diagnosis, and treatment of four horse patients, that had clinical symptoms and were simultaneously seropositive (3 horses positive in ELISA and one horse negative in ELISA, but positive in Immunoblot).

Patient	Age (Years) and Sex	Breed and Usage	Clinic and Therapy	Serology
1	3 y, F	Arabian, recreational riding	Hospitalised in March 2009 with a history of painful joint swelling of all limbs, lasting 14 days without reaction to the therapy. The mare was limping and had a fever (38.8–39.4 °C). The animal had a tiny nodule on the right side of her neck, suspected after insect bites. Treatment with cephalosporin antibiotics was without effect. Long-term therapy with broad-spectrum antibiotics and corticosteroids alleviated the clinical symptoms, but they resumed after discontinuation.	First serological examination by ELISA in 2009 was negative. Another examination in 2013 by Immunoblot (on three different microtiter plates) was positive, confirming Lyme disease.
2	2 y, F	Irish Cob, planned to be used for recreational riding.	Hospitalised in 2013 for clinical symptoms including fever (39.4 °C), apathy, cough, mucous discharge from the nostrils, tarsal joints’ swelling, limb pain, limping and unwillingness to move. Ticks or signs after insect bites were not found on her body. The animal did not receive any anti-borreliosis therapy, and due to serious health complications, was euthanised.	First serological examination in August 2013 was negative in ELISA for both IgM and IgG antibodies. Two months later, the horse was positive in ELISA for IgM antibodies.
3	8 y, castrate	Thoroughbred, recreational riding	Hospitalised in 2012 for clinical symptoms of central neuropathy, e.g., uncertain movement, apathy, head-turning to the left with shaking and jerking movement and labium twitching. Treated for acute borreliosis. The horse recovered from the diseases.	Seropositive in ELISA for IgM antibodies.
4	3 y, M	Thoroughbred, sport	Owner reported exercise intolerance. Since there was a diagnosis of dorsal displacement of the soft palate (DDSP), laser therapy was performed to improve the condition of the horse.	Seropositive in ELISA for IgM and IgG antibodies. It seems that the horse had asymptomatic seroconversion.

F: female; M: male.

## Data Availability

The data that support the findings of this study are available on request from the corresponding author. The data are not publicly available due to privacy or ethical restrictions.
